# Inter-rater reliability in labeling quality and pathological features of retinal OCT scans: A customized annotation software approach

**DOI:** 10.1371/journal.pone.0314707

**Published:** 2024-12-18

**Authors:** Katherine Du, Stavan Shah, Sandeep Chandra Bollepalli, Mohammed Nasar Ibrahim, Adarsh Gadari, Shan Sutharahan, José-Alain Sahel, Jay Chhablani, Kiran Kumar Vupparaboina

**Affiliations:** 1 Department of Ophthalmology, University of Pittsburgh Medical Center, Pittsburgh, PA, United States of America; 2 Department of Computer Science, University of North Carolina at Greensboro, Greensboro, NC, United States of America; Akita University: Akita Daigaku, JAPAN

## Abstract

**Objectives:**

Various imaging features on optical coherence tomography (OCT) are crucial for identifying and defining disease progression. Establishing a consensus on these imaging features is essential, particularly for training deep learning models for disease classification. This study aims to analyze the inter-rater reliability in labeling the quality and common imaging signatures of retinal OCT scans.

**Methods:**

500 OCT scans obtained from CIRRUS HD-OCT 5000 devices were displayed at 512x1024x128 resolution on a customizable, in-house annotation software. Each patient’s eye was represented by 16 random scans. Two masked reviewers independently labeled the quality and specific pathological features of each scan. Evaluated features included overall image quality, presence of fovea, and disease signatures including subretinal fluid (SRF), intraretinal fluid (IRF), drusen, pigment epithelial detachment (PED), and hyperreflective material. The raw percentage agreement and Cohen’s kappa (κ) coefficient were used to evaluate concurrence between the two sets of labels.

**Results:**

Our analysis revealed κ = 0.60 for the inter-rater reliability of overall scan quality, indicating substantial agreement. In contrast, there was slight agreement in determining the cause of poor image quality (κ = 0.18). The binary determination of presence and absence of retinal disease signatures showed almost complete agreement between reviewers (κ = 0.85). Specific retinal pathologies, such as the foveal location of the scan (0.78), IRF (0.63), drusen (0.73), and PED (0.87), exhibited substantial concordance. However, less agreement was found in identifying SRF (0.52), hyperreflective dots (0.41), and hyperreflective foci (0.33).

**Conclusions:**

Our study demonstrates significant inter-rater reliability in labeling the quality and retinal pathologies on OCT scans. While some features show stronger agreement than others, these standardized labels can be utilized to create automated machine learning tools for diagnosing retinal diseases and capturing valuable pathological features in each scan. This standardization will aid in the consistency of medical diagnoses and enhance the accessibility of OCT diagnostic tools.

## Introduction

Retinal diseases, encompassing conditions like age-related macular degeneration (AMD) and diabetic retinopathy (DR), represent significant contributors to global vision impairment and blindness. Optical coherence tomography (OCT) serves as the cornerstone imaging modality in ophthalmology imaging, leveraging light waves to capture detailed cross-sectional scans of the retina [1]. In particular, OCT scans play a pivotal role in diagnosing and guiding treatment decisions for various eye pathologies, thus contributing significantly to vision preservation.

Currently, the interpretation of OCT images by ophthalmologists relies heavily on their individual experiences and expertise, leading to variability in diagnoses. For instance, studies focusing on macular disease diagnosis and glaucoma detection using OCT have reported varying levels of interobserver agreement, underscoring the impact of individual interpretation on diagnostic consistency and treatment plans [2,3]. Specifically for AMD, a study showed significant variability in identifying complete retinal pigment epithelium and outer retinal atrophy (cRORA) between retina-trained ophthalmologists [4]. On the other hand, another study demonstrated substantial interrater agreement for qualitatively graded AMD features associated with atrophy, while other classification systems had varying amounts of agreement [5]. Other research using AMD OCT scans showed that a nine-point summary scale for grading may exhibit stronger intergrader agreement compared to binary grading [6].

As OCT data annotation by ophthalmologists requires specialist knowledge and is substantially time-consuming, there is a sparsity of labeled OCT datasets for a plethora of eye conditions including AMD. Traditionally, deep learning methods have required large amounts of labeled data to be used as input in the creation of effective algorithms. Recently, to combat the sparsity in labels for OCT data, studies have been conducted that enhance learning on limited data. For example, using a self-supervised learning phase followed by an OCT image classification learning phase, Fang et al. (2022) created a machine learning algorithm that can first learn inherent representations from the OCT images without manual labels which provided useful initialization parameters for the downstream OCT image classification model [7]. This tool classified features on OCT images, such as pigment epithelium detachment (PED), intraretinal fluid (IRF), subretinal fluid (SRF), normal retina, and retinal edema area (REA) with accuracies greater than 95% on the public RETOUCH dataset and greater than 98% on the AI Challenger dataset. While creating these promising techniques helps enhance learning on OCT data, a robust set of manually labeled images is still needed as a foundation for input as well as model evaluation in deep learning.

In response to current clinical challenges, the integration of artificial intelligence (AI) algorithms with OCT data offers promising avenues for improving early disease detection and expanding diagnostic capabilities, particularly in underserved regions [8–15]. Automated report generation using AI on OCT data represents a significant opportunity for enhancing diagnosis and treatment of retinal diseases, providing valuable decision-support tools for clinicians. Recent advancements have demonstrated the practical clinical application of AI algorithms in proficiently identifying pathologic retinal cases and efficiently triaging patients [16–23]. However, the efficacy of these algorithms relies heavily on accurate labeling of pathological features by medical specialists, highlighting the importance of evaluating inter-rater reliability in OCT scan interpretations to establish robust "gold-standard" labels [24,25].

Moreover, the scarcity of labeled OCT datasets for various eye conditions poses a significant challenge for traditional deep learning methods, which typically require large amounts of labeled data for effective algorithm creation [26–30]. Recent studies have explored innovative approaches to address this challenge, such as self-supervised learning phases followed by OCT image classification learning phases [7,31–35]. These efforts aim to enhance learning on limited data and improve the performance of AI algorithms in diagnosing retinal diseases. Additionally, the quality of OCT scans significantly impacts the performance of machine learning algorithms [36–39]. Variations in image quality can affect the accuracy of diagnoses, as evidenced by studies focusing on retinopathy screening in infants. Strategies for enhancing image quality, such as deep learning-based image quality assessment and enhancement systems, have shown promising results in improving diagnostic accuracy [40–43].

The objective of this study is to evaluate the inter-rater reliability of OCT scan interpretation for AMD eyes among independent reviewers, specifically focusing on labeling for scan quality and pathological retinal features. We aim to assess the ability of independent reviewers to reach a consensus on annotating different aspects of OCT B-scans with AMD. We believe this work is a steppingstone towards generating standardized ground-truth labeled data for building AI-based diagnostic tools and in turn enhance the accuracy of disease screening in clinical practice.

## Methods

### Dataset

This retrospective study was conducted in accordance with the principles outlined in the Declaration of Helsinki, with approval from the institutional review board of the University of Pittsburgh Medical Center (UPMC), Pittsburgh. Informed consent was obtained as written consent from all participants for the inclusion of their retrospective data in the study. We utilized 500 OCT volumes obtained from the Cirrus 5000 OCT device (Carl Zeiss Meditec) corresponding to individual subjects diagnosed with age-related macular degeneration (AMD). Each Cirrus OCT volume comprised 128 B-scans, with a lateral resolution of 6mm and a depth resolution of 2mm, resulting in a pixel resolution of 512 × 1024. To yield a total of 500 B-scans for analysis, 16 B-scans were uniformly sampled from each volume until reaching this count, totaling the inclusion of 32 unique eyes. Patients were screened between 2017 and 2019. This data was accessed on May 31, 2023 for research purposes, and authors did not have access to information that could identify individual participants during or after data collection.

### Feature description

As image quality of OCT scans may affect diagnostic interpretations [44,45], it is critical to evaluate alongside labeling scans with pathological features. In this study, image quality was quantified as good, usable, or bad. If the scan exhibited bad quality, the reasons for poor quality were specified as speckle noise, artifacts, low contrast, or cropping. In the following, we describe the varying levels of scan quality considered for annotation:

***Good quality*:** All retinal sublayers are distinct, pathological features can be clearly observed.

***Usable quality*:** Some clarity lacking so that there may be slight blur or contrast issues, but most pathological features can be observed.

**Fig 1:**
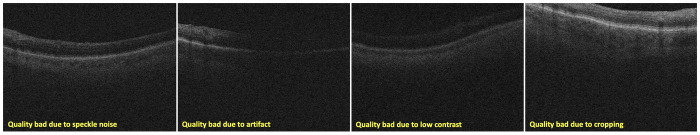
Examples of labels for reason for poor quality scan: Speckle noise, artifact, low contrast, and cropping.

***Bad quality*:** Retinal sublayers cannot be clearly seen or are covered. If scan falls in this category, the reason for poor quality is classified as follows (Fig 1):

Speckle noise: Speckle noise in OCT images is a granular pattern caused by random light scattering and interference, reducing image clarity and obscuring fine details [46]. It makes it difficult for clinicians to visualize retinal structures and corresponding structural changes and can hinder accurate detection of retinal abnormalities.

Artifact: Artifacts in OCT images often happen when a patient blinks or moves their eyes during the scan. Blinking can create gaps or missing parts in the image, while movement causes blurring or misalignment of the retinal layers. These distortions can make it harder to detect and analyze disease features accurately [47].

Low contrast: A bad quality OCT image due to low contrast occurs when the differences in intensity between adjacent structures or layers in the eye are too subtle for clear visualization [48]. Low contrast can make it difficult to distinguish critical features, such as retinal layers or disease markers like drusen and fluid. This results in a "washed-out" appearance, where boundaries between structures are blurred, reducing the clarity of important diagnostic details. Factors contributing to low contrast include poor illumination, scattering of light, media opacities in the eye (like cataracts), or suboptimal OCT device settings.

Cropping: A side of the retinal tissue layers in a scan is cut off, so that the image is clipped to a smaller dimension and part of the scan is not visible.

**Fig 2:**
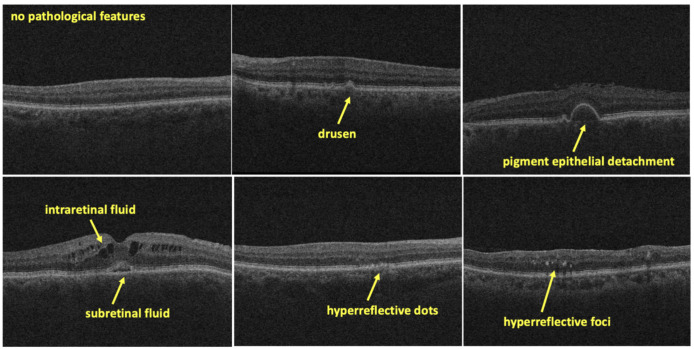
Examples of labels for drusen, pigment epithelial detachment, intraretinal fluid, subretinal fluid, hyperreflective dots, and hyperreflective foci.

Retinal OCT scans provide detailed in vivo visualization of the structural changes of the posterior segment substructures including retinal sublayers, capturing various pathological features indicative of retinal diseases. This study aims to identify features that are associated with AMD disease. Fig 2 presents the representative OCT scans depicting various disease manifestations of AMD disease. In the following, we describe various AMD disease signatures considered for annotation:

***Subretinal Fluid (SRF)*:** SRF refers to the accumulation of fluid between the retina and the retinal pigment epithelium (RPE) layer in the eye. Normally, the subretinal space is devoid of fluid, but in various retinal diseases and conditions, fluid can accumulate in this space, leading to SRF. In the OCT image, they appear as dark spaces between the RPE and the neurosensory retina [49].

***Intraretinal Fluid (IRF)*:** IRF refers to the accumulation of fluid within the layers of the retina itself. Unlike subretinal fluid, which accumulates between the retina and the RPE layer, IRF initially presents as diffuse thickening of the outer nuclear layer of the retina which if more severe may form cystoid spaces that may involve all retinal layers [49]. IRF appears as dark spaces within the retinal layers.

***Pigment Epithelial Detachment (PED)*:** PED is a condition characterized by the accumulation of fluid or material underneath the RPE, appearing on OCT as elevations of RPE band relative to Bruch’s membrane [49].

***Drusen*:** Drusen are tiny yellow or white deposits that accumulate under the retina [50]. They are found in AMD eyes, but may also be a normal feature of aging eyes [51]. Drusen is composed of lipids and proteins that have been deposited in the blood [51].

***Hyperreflective Dots*:** Small, bright reflections within the retinal layers, often indicating cellular debris or inflammatory cells [52].

***Hyperreflective Foci*:** Larger, more distinct bright spots within the retinal layers, associated with retinal inflammation, ischemia, or neovascularization [53,54].

In early AMD, drusen and hyperreflective dots are key features present on OCT scans [55,56]. In intermediate AMD, drusen, PED, IRF, and hyperreflective foci are prevalent on OCT scans [57–59]. In late AMD, pathological features observed include drusen, PED, IRF, SRF, hyperreflective dots, and hyperreflective foci [60,61]. Many of these features may also be present in other retinal diseases such as diabetic retinopathy, central serous chorioretinopathy, and retinal vein occlusion.

### Annotation tool

**Fig 3:**
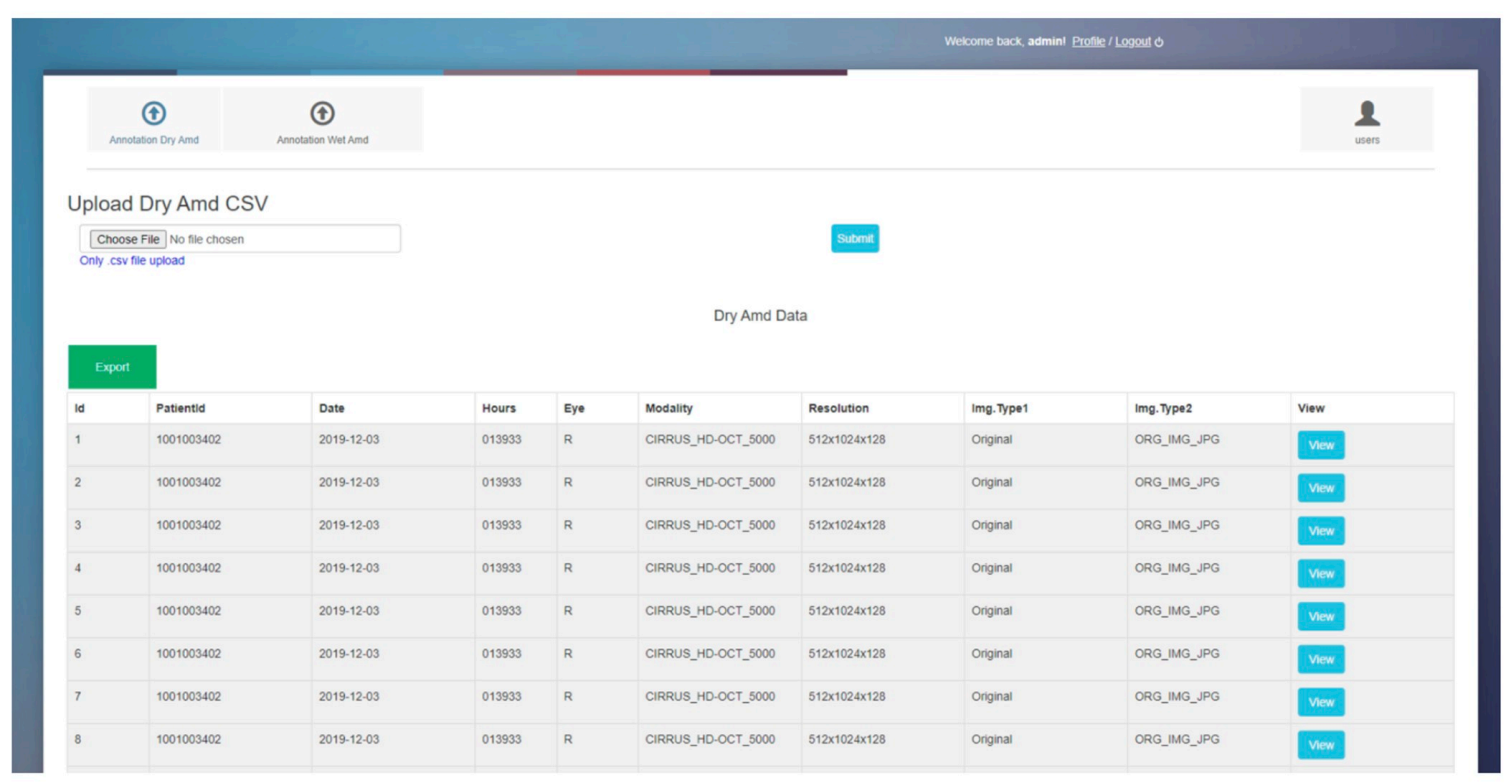
Home page of OCT image labeling software created and used for labeling OCT scans.

**Fig 4:**
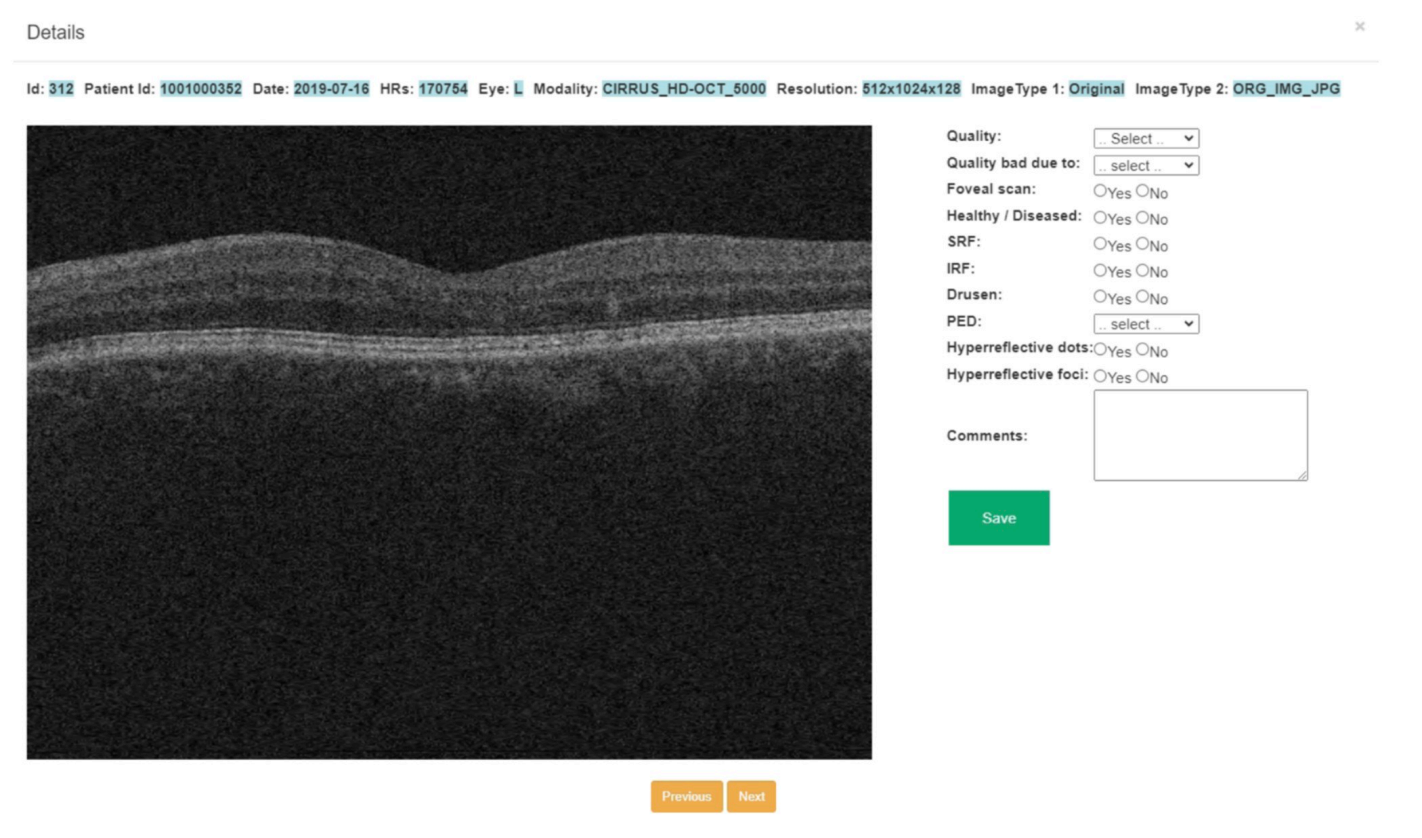
Interactive window pop-up of OCT scan used to label a single scan.

We developed OCT image labeling software in-house for annotating retinal features on scans (Fig 3). This software, created using PHP and SQL platforms, was deployed as a standalone application using an Apache server. The software enables users to create separate accounts and independently label a set of OCT scans. Accordingly, we created separate accounts for two reviewers (Katherine and Stavan) for performing the annotations. The interface presents a table on the home screen listing each OCT scan along with its relevant characteristics, including patient research ID, laterality (right or left eye), imaging modality, resolution, and image type. There is a “View” button corresponding to each B-scan that reviewers can select to perform the annotation. Clicking the “View” button takes the reviewer to a different page where the reviewer can view the full view of the B-scan with the list of features to annotate on the right of the image (Fig 4). There are check boxes for “Yes” or “No” for binary classification i.e., for annotating the presence or absence of certain disease features. Further, there are also dropdown menus for some aspects of annotation including reason for scan quality and type of PED. Accordingly, the reviewer annotates each OCT image either by selecting options from the drop-down or by checking the check boxes for all aspects of the OCT image including scan quality, reason for poor quality, presence of specific retinal features, and additional comments. Once an image is labeled by a reviewer, the table updates the scan status from unlabeled to labeled. Annotations are saved, and reviewers can proceed to the next OCT scan. The aggregated data of all labels can be downloaded for further analysis.

### Annotation strategy

Two medical students (Katherine Du and Stavan Shah) underwent training by a retinal ophthalmologist (Jay Chhablani) to independently label the OCT B-scan features under consideration. In particular, the medical students separately reviewed a range of OCT scans to gauge scan quality as well as several examples of each disease feature to be labeled. The medical students next trained one-on-one with the retinal ophthalmologist to label sample scans, then worked together to label other sample scans to further solidify their understanding of these features. Lastly, the retinal ophthalmologist reviewed the initial 50 OCT scans in the official dataset independently labeled by the medical students to ensure their validity, and also provided clarifications on any of the 500 scans in which the medical students had uncertainty.

**Fig 5:**
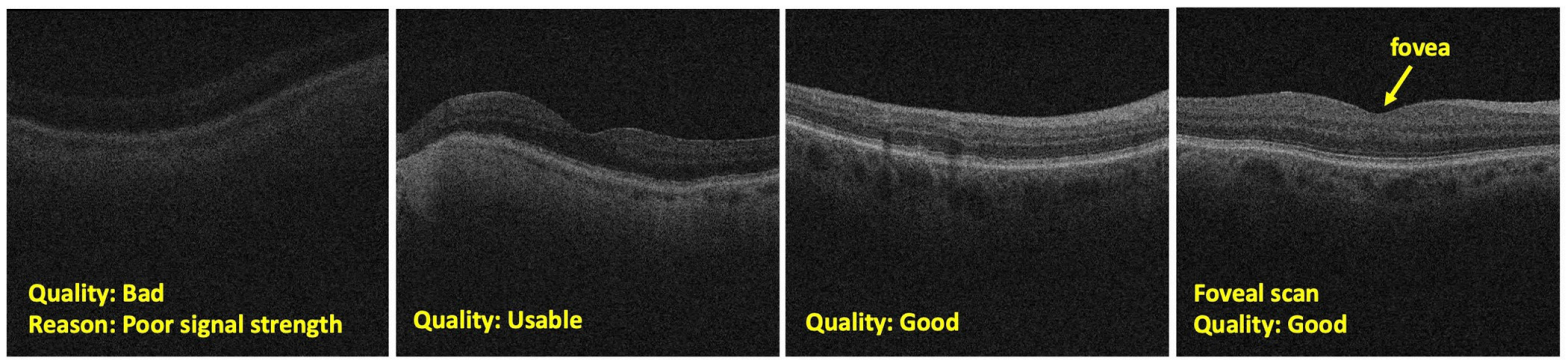
Examples of labels for overall quality (good, bad, useable), reasons for poor quality, and foveal scan.

In the labeling process, first, the scan quality was determined as either good (clear), usable (identifiable but not optimal), or poor (unidentifiable) (Fig 5). Further, the reasons for poor-quality scans were classified as speckle noise, artifacts, low contrast, or cropping (Fig 5). Next, reviewers grade if the scan is a foveal scan or a non-foveal scan (Fig 5). Subsequently, reviewers look for the presence and absence of the disease features on OCT scans and grade them as healthy (scans with no retinal disease signature) or diseased (scans with retinal disease signatures). If the scan is labeled as diseased, then reviewers proceed to annotate for the presence or absence of specific disease signatures including subretinal fluid, intraretinal fluid, drusen, PED, hyperreflective dots, and hyperreflective foci. Further, if PED was present, the type of PED was indicated as fibrovascular, flat irregular, serous, drusenoid, or hemorrhagic. The reviewers also are provided with a “comments” box to make any specific comments about the image under consideration. This comprehensive annotation strategy aimed to capture the full spectrum of retinal characteristics and disease manifestations present in the OCT scans.

### Statistical analysis

To assess inter-observer variance, raw percentage agreement was calculated for each feature. Additionally, Cohen’s kappa (κ) coefficient was computed as a measure of inter-rater reliability [62]. Kappa values range from –1 to 1, with < 0 indicating worse than chance agreement, 0.01–0.20 indicating slight agreement, 0.21–0.40 indicating fair agreement, 0.41–0.60 indicating moderate agreement, 0.61–0.80 indicating substantial agreement, and 0.81–1.00 indicating almost perfect agreement. Both percentage agreement and kappa statistics were utilized as metrics in this study.

## Results

**Fig 6:**
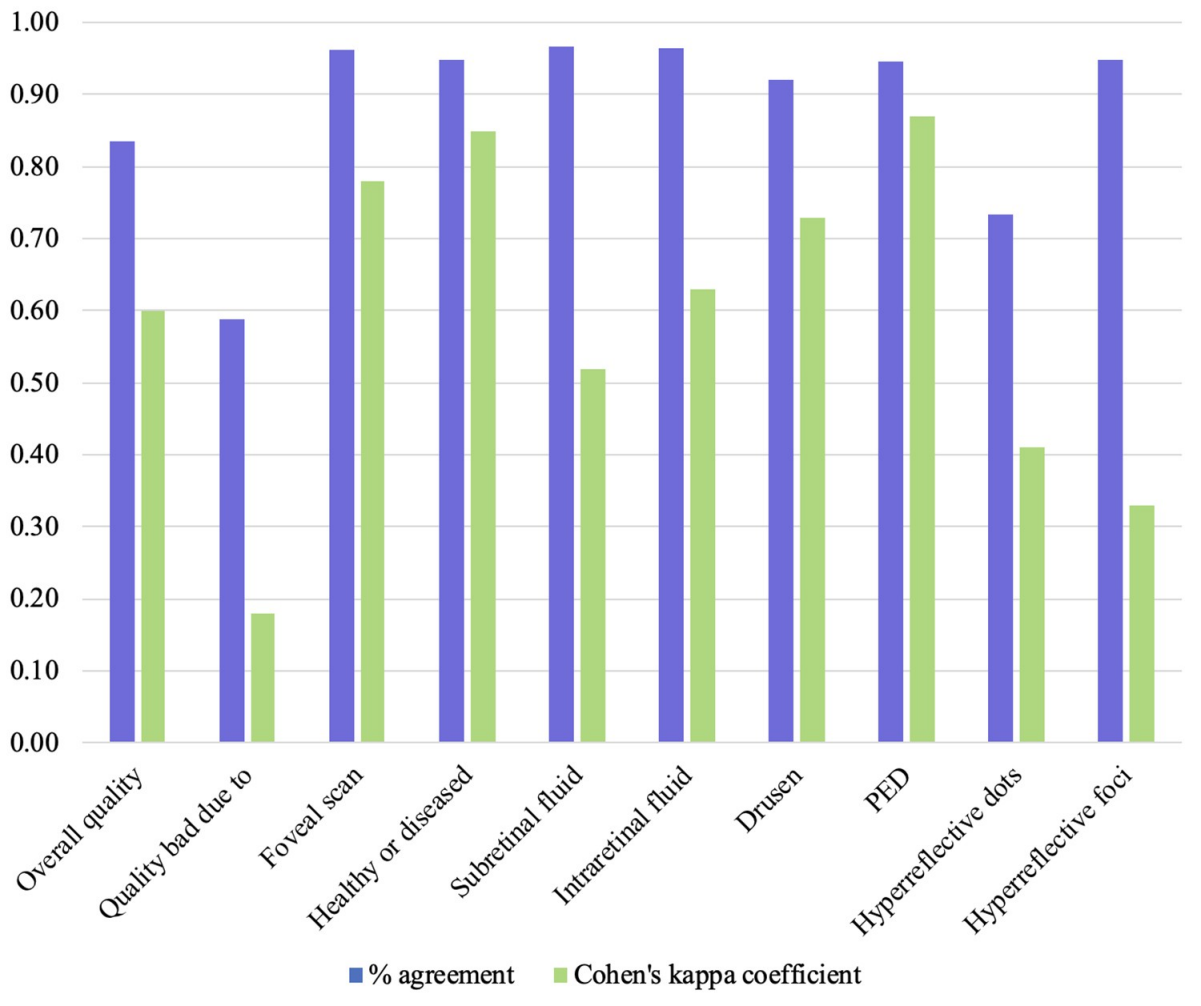
Inter-rater reliability for 500 OCT scans, reported as raw percentage match and Cohen’s kappa coefficient. blue = % match, green = Cohen’s kappa coefficient.

The results of the inter-rater reliability analysis for labeling OCT retinal scans revealed significant agreement between independent reviewers for various pathological features and scan quality assessments (Fig 6).

Scan Quality Agreement: The agreement between reviewers regarding the overall quality of OCT scans as bad, usable, or good was moderate to substantial, with a Cohen’s kappa coefficient (κ) of 0.60 (raw percent agreement of 84%). This indicates a consistent assessment of scan quality, which is crucial for ensuring the reliability of subsequent analyses and machine learning algorithms.

**Fig 7:**
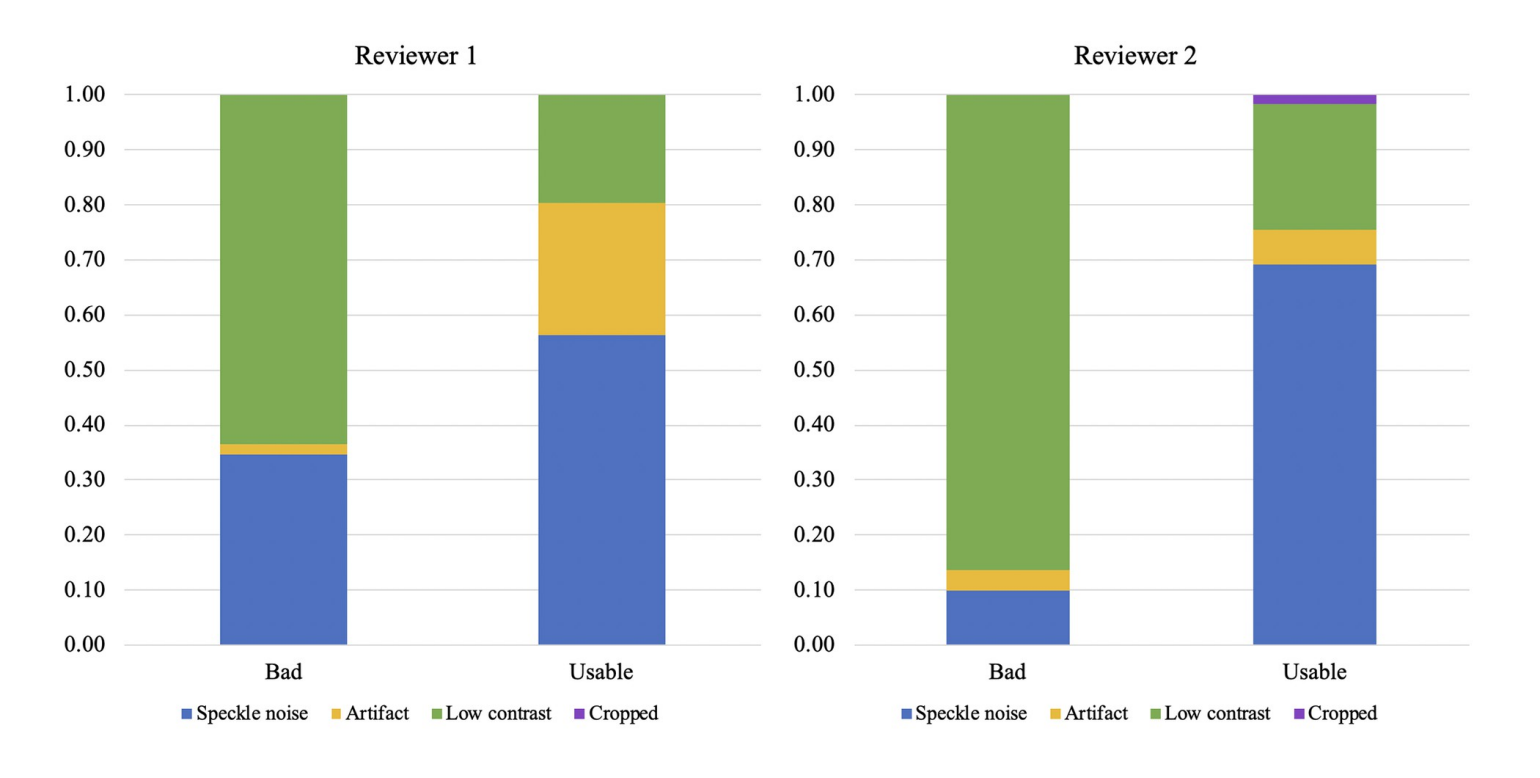
Percent breakdown of reasons for poor quality in bad and usable scans independently labeled by reviewer 1 and reviewer 2. Total number of bad quality scans by reviewer 1 and reviewer 2 were 52 and 51, respectively. Total number of usable quality scans by reviewer 1 and reviewer 2 were 87 and 65, respectively. blue = speckle noise, green = low contrast, yellow = artifact, purple = cropped.

Reasons for Poor Quality Scan Agreement: The reasons for poor quality on a scan showed slight agreement, with a κ coefficient of 0.18 (raw percent agreement of 59%). For usable and bad scans, where quality affects the ability to observe retinal sublayers or pathological features, the breakdown of reasons for poor quality are presented in Fig 7. For both reviewers, the majority of OCT scans labeled as “bad quality” were due to low contrast in the scan; reviewer 1 graded more than 60% of bad scans as due to low contrast, while reviewer 2 graded more than 80% of bad scans as due to low contrast. Speckle noise was present in both bad and useable scans, with a higher percentage of usable scans exhibiting speckle noise (about 55% of usable scans graded by reviewer 1, about 70% of usable scans graded by reviewer 2). Artifacts occurred less frequently than speckle noise for both reviewers and are present in both bad and useable scans. Lastly, cropped images are sparsely represented, making up none of the scans of reviewer 1 and a slight percentage of the usable scans of reviewer 2.

Pathological Features Agreement: Regarding binary grading scans for the presence and absence of diseased signatures, the inter-rater reliability was almost in perfect agreement, with a κ coefficient of 0.85 (raw percent agreement of 95%). This high level of agreement suggests robust consistency in identifying scans without disease signatures in retinal layers of the OCT B-scans.

Among specific retinal pathologies, substantial to almost perfect agreement was observed for several features. The presence of a foveal scan exhibited a κ coefficient of 0.78 (raw percent agreement of 96%), indicating strong concurrence between the reviewers. Similarly, agreement was substantial for identifying PED type, with a κ coefficient of 0.87 (raw percent agreement of 95%). Moderate to substantial agreement was found for subretinal fluid, intraretinal fluid, and drusen, with κ coefficients of 0.52, 0.63, and 0.73, respectively. However, some features showed weaker agreement between the reviewers. In particular, the κ coefficients for identifying hyperreflective dots was 0.41 and hyperreflective foci was 0.33. Although these features still demonstrated fair agreement, there was slightly more variability in their interpretation compared to other features.

## Discussion

**Fig 8:**
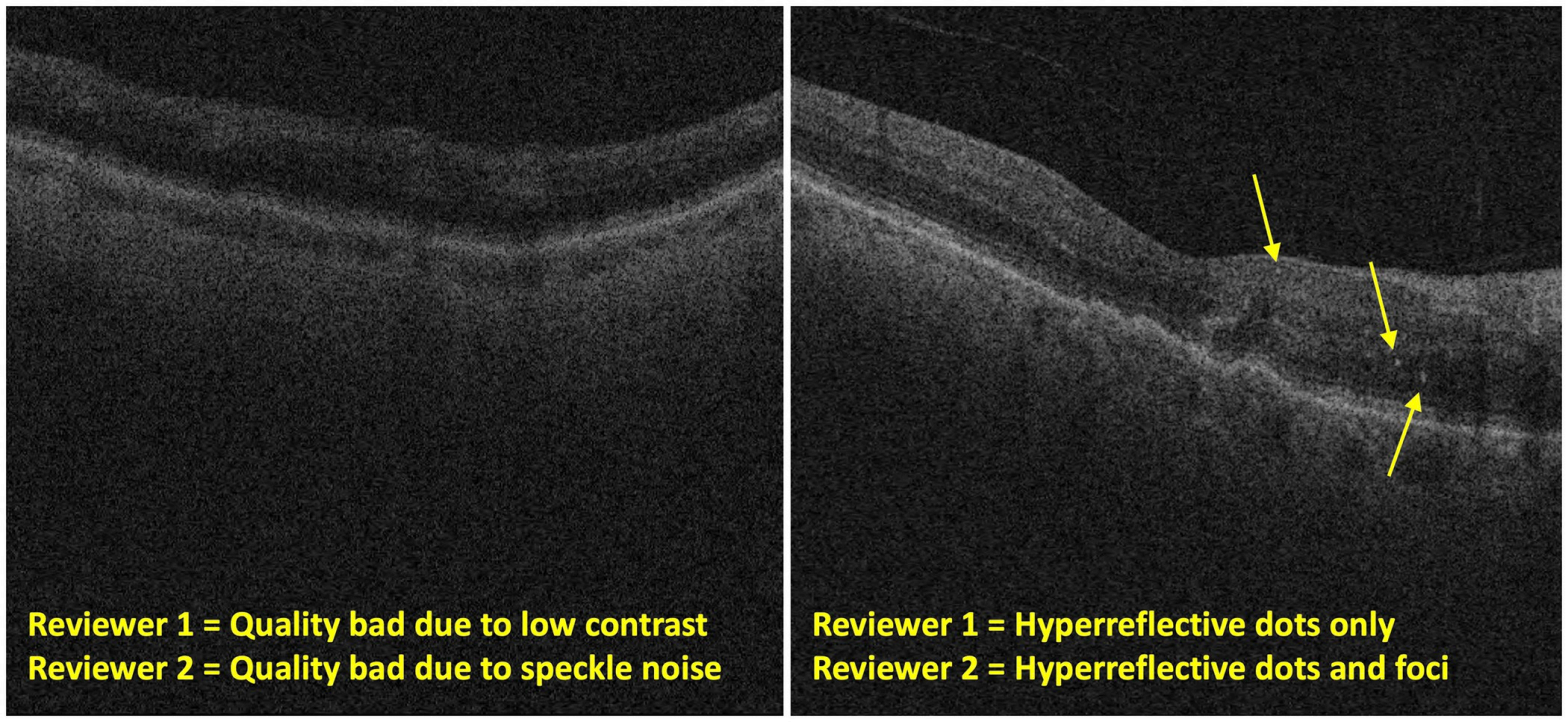
Disagreements between reviewers for features in OCT scans. (L) disagreements in reason for bad quality (R) disagreements in presence of hyperreflective material.

Our study shows that there is significant inter-rater reliability in labeling the quality and retinal pathologies on OCT scans of the retina. As the quality of scans is an important determinant for clinical decision-making and building AI-based tools for automated detection of disease signatures, the considerable agreement between the two reviewers indicates that scans deemed better quality can be exclusively used or weighed more in models to guarantee more accurate model learning. However, the reason for poor quality scan differs between the reviewers, indicating that there may be multiple factors contributing to an unclear scan which leaves more interpretation up to the reviewer. This indicates that it may also be difficult for AI models to determine a singular cause for poor-quality scans. For example, some of the OCT scans exhibited multiple reasons for quality deficits, such as speckle noise and low contrast, and reviewers were asked to indicate the one predominant reason for poor quality (Fig 8). These differences could have led to the lower κ coefficient of 0.18 for the “quality bad due to” category, while the “overall quality” of good, useable, or bad received higher concurrence with κ = 0.60. However, some common themes arise in the “quality bad due to” category, as both reviewers labeled “low contrast” as the predominant reason for a bad quality scan (60% by reviewer 1, 80% by reviewer 2). This indicates that low contrast may be a key factor impacting scan quality and the interpretability of the OCT scan for pathological features assessment. Hence, machine learning algorithms that read OCT scans may increase their accuracy by excluding low contrast scans, or by learning to differentiate these low contrast scans and interpreting the scan differently than it would with a good quality scan. As differing amounts of speckle noise is present in most OCT scans, if artifact, low contrast, or cropping were not present, the default reason for a poor-quality scan was mostly speckle noise. Artifacts seem to be minimally present in scans with bad or usable quality, with cropping occurring even less. Hence, the overarching reason for poor image quality that may result in unusable or incorrectly interpreted scans in downstream analysis is low contrast due to the OCT imaging collection process.

In terms of pathological features of the retina, there is almost perfect agreement in making binary decision about whether the scan has the presence or absence of the disease signatures. This requires the synthesis of information about the identification of several other pathological features to come to a determination. Since these individual features (SRF, IRF, drusen, PED, hyperreflective dots, hyperreflective foci) also have excellent concurrence between the two reviewers, it strengthens the diagnosis of healthy or diseased eye by providing the grounds for this interpretation. There was also strong concurrence for the specific PED out of the five types, with a κ coefficient of 0.87 for the 500 scans. Thus, we have stronger confidence that this feature is standardized amongst independent reviewers and can readily be used in future AI models. As for hyperreflective dots and foci, their κ coefficient was 0.41 and 0.33, indicating a weaker match. This could be explained by the fact that many of the OCT scans had hyperreflective material that differed in size and shape which the reviewers may have interpreted differently (Fig 8). Specifically, the relatively smaller size of hyperreflective dots and the relatively larger size of hyperreflective foci is on a spectrum, and up to the qualitative judgment of reviewers as to its presence in a scan. Additionally, some scans exhibit minimal hyperreflective material, and the reviewer determines to what extent it is significant enough to quantify. Hence, hyperreflective dots and foci could be grouped together into a single feature for AI models or each distinct feature not weighed as significantly. Lastly, foveal scan has strong agreement as well, with a κ coefficient of 0.78. As retinal pathologies in the fovea may cause more noticeable visual impairment, identifying the presence of a foveal scan plus pathological features or the fact that the layers of the retina are more curved on foveal scans and distort the appearance of pathological features may be key in accurate triage or diagnosis using OCT scans.

Limitations to this study include the particular scope of OCT scans used for concurrence analysis, which was capturing age-related macular degeneration eyes with CIRRUS HD-OCT 5000 imaging devices. Hence, the assessment of quality and pathological retinal features of other eye diseases such as diabetic retinopathy and central serous chorioretinopathy can be further explored. Additionally, quality of scans and reasons for poor quality scans may be examined on other OCT imaging devices, such as Heidelberg Engineering SPECTRALIS OCT or Topcon 3D OCT-2000. Lastly, on the annotation software, reviewers added comments onto individual OCT scans to indicate the presence of other retinal signatures that were not explicitly listed. However, the interrater reliability of these other retinal signatures, such as epiretinal membrane and geographic atrophy, can be analyzed in future studies.

These standardized labels can be used to create automated algorithms that help standardize medical diagnoses and increase the accessibility of OCT diagnostic tools. Firstly, OCT image quality should be taken into consideration, as our study demonstrates that low contrast is a common reason for a poor-quality scan, which may lead to limited readability or diagnostic misinterpretation by machine learning algorithms. Either excluding poor quality scans or training machine learning models to interpret these scans differently may alleviate downstream analysis issues resulting from varying scan quality. Our study demonstrates that labels created for retinal pathological features are generally agreeable between reviewers, with some features displaying stronger concurrence compared to others. This indicates that there may be slight variability in ophthalmologists’ diagnosis of retinal diseases using OCT images, and that supporting OCT information with patient records may present a more definite diagnosis. Additionally, it shows us which features may have stronger diagnostic capacity, such as PED, subretinal fluid, and intraretinal fluid, compared to others. The widespread use of electronic medical records, with deep neural networks in machine learning, allows for the adaptation of artificial intelligence image analysis tools for computer-aided diagnosis. The conclusions from this analysis can be integrated into engineering decisions in the creation of these AI models for the diagnosis of retinal diseases.
